# Inter-reader agreement of the prostate imaging reporting and data system version v2.1 for detection of prostate cancer: A systematic review and meta-analysis

**DOI:** 10.3389/fonc.2022.1013941

**Published:** 2022-09-29

**Authors:** Jing Wen, Yugang Ji, Jing Han, Xiaocui Shen, Yi Qiu

**Affiliations:** ^1^ Department of Medical Imaging, Jiangsu Vocational College of Medicine, Yancheng, China; ^2^ The First People’s Hospital of Yancheng, The Fourth Affiliated Hospital of Nantong University, Yancheng, China; ^3^ The Affiliated Suzhou Science & Technology Town Hospital of Nanjing Medical University, Suzhou, China

**Keywords:** prostate cancer, magnetic resonance imaging, inter-reader agreement, meta-analysis, PI-RADS

## Abstract

**Objectives:**

We aimed to systematically assess the inter-reader agreement of the Prostate Imaging Reporting and Data System Version (PI-RADS) v2.1 for the detection of prostate cancer (PCa).

**Methods:**

We included studies reporting inter-reader agreement of different radiologists that applied PI-RADS v2.1 for the detection of PCa. Quality assessment of the included studies was performed with the Guidelines for Reporting Reliability and Agreement Studies. The summary estimates of the inter-reader agreement were pooled with the random-effect model and categorized (from slight to almost perfect) according to the kappa (*κ*) value. Multiple subgroup analyses and meta-regression were performed to explore various clinical settings.

**Results:**

A total of 12 studies comprising 2475 patients were included. The pooled inter-reader agreement for whole gland was *κ*=0.65 (95% CI 0.56-0.73), and for transitional zone (TZ) lesions was *κ*=0.62 (95% CI 0.51-0.72). There was substantial heterogeneity presented throughout the studies (*I* ^2^= 95.6%), and meta-regression analyses revealed that only readers’ experience (<5 years vs. ≥5 years) was the significant factor associated with heterogeneity (P<0.01). In studies providing head-to-head comparison, there was no significant difference in inter-reader agreement between PI-RADS v2.1 and v2.0 for both the whole gland (0.64 vs. 0.57, *p*=0.37), and TZ (0.61 vs. 0.59, *p*=0.81).

**Conclusions:**

PI-RADS v2.1 demonstrated substantial inter-reader agreement among radiologists for whole gland and TZ lesions. However, the difference in agreement between PI-RADS v2.0 and v2.1 was not significant for the whole gland or the TZ.

## Introduction

Prostate cancer is the second most common cancer in men worldwide, and it is estimated that one in nine will be affected by this disease at some point during their lifetime (1, 2). Multiparametric magnetic resonance imaging (mpMRI) has been established as an effective noninvasive method for the detection, staging, and guiding management of PCa (3–5). Compared with conventional methods that depend on digital rectal examination (DRE) or transrectal ultrasonography (TRUS)–guided biopsy alone, mpMRI demonstrated higher accuracy and thus could substantially reduce unnecessary biopsies (6–8). In recent years, various novel models and nomograms were developed to improve the diagnostic performance of PCa, which utilizes one or more of following tools or biomarkers: serum prostate-specific antigen density (PSA), PSAD, age, history of prior prostate biopsy, DRE, radiomics, genomics, and molecular imaging (9, 10)

To standardize performing, interpreting, and reporting the PCa with mpMRI, the European Society of Urogenital Radiology (ESUR) introduced the first version of the Prostate Imaging Reporting and Data System in 2012, which was generated based on expert consensus and provides a detailed scoring system using mpMRI (11). The PI-RADS has been validated as an effective scoring system in clinical practice, and an early meta-analysis reported that the pooled sensitivity and specificity for the first version were 0.78 and 0.79 (12). To address some shortcomings in the PI-RADS v1, the American College of Radiology (ACR) and the ESUR released PI-RADS v2, which was widely applied for risk stratification and determination of biopsy pathway, showing good diagnostic performance (13, 14). However, inter-reader agreement among radiologists for PI-RADS v2 varied widely, especially for TZ lesions (15, 16). Therefore, a revision termed PI-RADS v2.1 was released in 2019, which primarily aimed to improve the inter-reader agreement on TZ (17). The major revisions are as follows: 1) introducing the concept of atypical nodules to score 2 in TZ, which are defined as a mostly encapsulated or homogeneous circumscribed nodules; 2) employing the DWI features to upgrade atypical nodules to score 3; and 3) downgrading these completely encapsulated nodules to score 1. Several studies evaluating the reproducibility of PI-RADS v2.1 especially for TZ have been published; however, the reported inter-reader agreements varied widely. Therefore, the purpose of our study was to systematically assess the reproducibility of PI-RADS v2.1 among various radiologists. Besides, we aimed to compare the inter-reader agreement of PI-RADS v2.1 with PI-RADS v2 in studies providing head-to-head comparison.

## Methods

This meta-analysis and systematic review is reported in accordance with the Preferred Reporting Items for Systematic Reviews and Meta-Analysis (PRISMA) statement, and performed with a predefined review and data extraction protocol (18). The primary outcome of our study was the pooled inter-reader agreement of using the PI-RADS v2.1 for the prediction of PCa. As the primary revision in PI-RADS v2.1 was the improvement of reproducibility for TZ lesions, the secondary outcome of our study was the inter-reader agreement regarding TZ. In addition, we would compare the PI-RADS v2.1 and v2.0 in studies providing head-to-head comparison.

### Search strategy and selection criteria

A computerized literature search of PubMed, EMBASE, Cochrane Library, Web of Science, and Google Scholar was performed to identify potential eligible articles published between September 1, 2019 and March 31, 2022. We included studies reporting the inter-reader agreement on PI-RADS v2.1, with no language restriction applied. The terms combined synonyms used for searching were as follows: ([PI-RADS] OR [Prostate Imaging Reporting and Data System]) AND ([prostate cancer] OR [prostate carcinoma] OR [PCa]). An additional search was performed by manually screening the bibliographies of the included articles and reviews. Studies identified by the literature search were assessed by two independent reviewers (*W.J.* with 6 years and *J.Y.G.* with 8 years of experience in performing systematic reviews and meta-analyses), and disagreements were resolved by consensus *via* discussion with a third reviewer (*Q.Y.*).

### Inclusion and exclusion criteria

We included studies that satisfied all of the following criteria: 1) reported the inter-reader agreement of PI-RADS v2.1 for detection of PCa; 2) provided *κ*values and 95% confidence intervals (95% CI), or other measurements allowing assessment of inter-reader agreement; and 3) reported TRUS-guided biopsy, MRI-US fusion biopsy, or radical prostatectomy (RP) pathological results as the reference standard. We excluded studies that met any of the following criteria: 1) studies with a small sample size that involved less than 20 participants; 2) studies that did not provide detailed data to evaluate the inter-reader agreement; and 3) reviews, letters, guidelines, conference abstracts, or editorials.

### Data extraction and quality assessment

We used a predefined standardized form to extract relevant information as follows: 1) demographic characteristics such as the number of patients and lesions, patient age, PSA level, and zonal anatomy (peripheral zone [PZ] and/or TZ); 2) study characteristics such as first author, publication year, location and institution, period of the study conducted, number of readers and their experience, whether blinded to final results, reference standard, inter-reader agreement; and 3) technical characteristics such as magnetic field strength, coil type, and MRI sequences used. The quality assessment of included studies was performed according to the Quality Appraisal of Diagnostic Reliability (QAREL) Checklist (19). For individual studies, these categories were scored as high quality if it was described in sufficient detail in the article with no potential bias.

### Data synthesis and statistical analysis

The summary estimates of inter-reader agreement values were calculated with the random-effects model *(*Sidik-Jonkman method) (20, 21), and then categorized as follows: a *κ* value of <0.20 indicates slight agreement; a *κ* value between 0.21 and 0.40, fair agreement; a *κ* value between 0.41 and 0.60, moderate agreement; a *κ* value between 0.61 and 0.80, substantial agreement; and a *κ* value of between 0.81 and 1.00, almost perfect agreement (22).

Aside from the grouping of all PI-RADS category 1-5 lesions, we performed multiple subgroup analyses regarding the following variables: 1) for PI-RADS category ≥3; 2) for PI-RADS category ≥4; 3) for lesions of TZ; 4) for lesions of the whole gland; 5) for readers with experience at least 5 years (experienced); 6) for readers with experience less than 4 years or not dedicated in this area (inexperienced). Considering that several studies provide head-to-head comparison between PI-RADS v2.1 and v2.0, we thus compared these two versions in available studies. The meta-regression analysis was performed to explore the causes of heterogeneity by adding the following covariates: 1) type of analysis (per patient vs. per lesion); 2) PI-RADS score (for lesions with score ≥3 vs. all lesions; 3) zonal anatomy (whole gland vs. TZ); and 4) readers’ level of experience (experienced vs. varied experience).

Heterogeneity throughout studies was determined with the *Q* statistics and the inconsistency index (*I* ^2^) as follows: for value between 0% and 40%, unimportant; between 30% and 60%, moderate; between 50% and 90%, substantial; and between 75% and 100%, considerable (23). Funnel plots and the rank test were used for the assessment of any possible publication bias. All analyses were performed with STATA 16.0 (StataCorp, Texas, USA), with *P* values <0.05 considered statistically significant. Two reviewers (*W.J.* and *J.Y.G.*) independently conducted the data extraction and quality assessment, disagreements were resolved through discussion and arbitrated by the third one (*Q.Y.*).

## Results

### Literature search and data extraction

**Figure 1:**
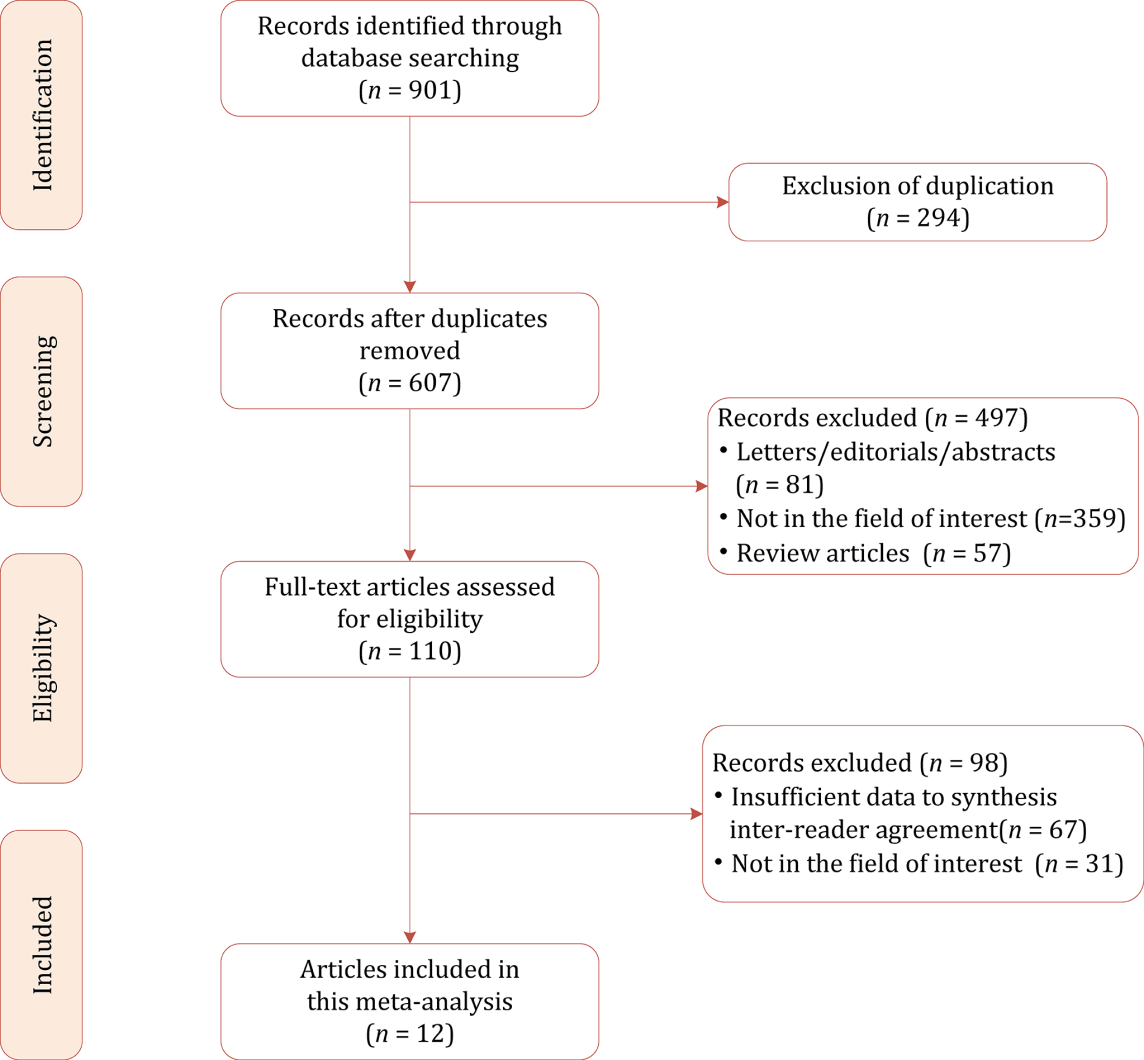
Study selection process for this systematic review and meta-analysis.

**Figure 1** shows the flow chart of the publication selection process. Our literature search identified 901 references initially, of which 294 were excluded due to duplicates. After inspecting the titles and abstracts, a total of 497 articles were excluded. The full-text review was conducted among the remaining 110 potential articles, of them 98 articles were excluded for insufficient data and not in the field of interest. Finally, a total of 12 articles comprising 2475 patients were included in the current meta-analysis (24–35).

### Characteristics of the included studies

The detailed demographic and study characteristics are summarized in **Tables 1**, **2**. The patient sample ranged from 58 to 355, with the lesion number of 58-638. The mean patient age was 63.1-73 years and with a PSA level of 4.9-13.7 ng/ml. Regarding zonal anatomy, 5 studies reported the inter-reader agreement only for TZ (27, 30, 31, 33, 34), 2 studies reported the inter-reader agreement both for TZ and PZ (24, 32), 4 studies reported the reproducibility for the whole gland and did not differentiate location (25, 26, 28, 35), only 1 study reported the inter-reader agreement merely on PZ lesions (29). In 4 studies the MRI images were interpreted by 2 radiologists (28, 29, 31, 34), whereas in the remaining 8 studies the MRI images were interpreted by at least 3 radiologists. Regarding readers’ experience, 5 studies reported that the MRI images were interpreted by experienced or dedicated radiologists (25, 27, 31, 34, 35), whereas in the remaining 7 studies the images were interpreted by radiologists with varied experience (1-20 years). With regard to magnetic field strength, in 10 studies the MRI examinations were performed with 3.0 T, while the remaining 2 studies used 1.5 T MRI (25, 26). Endorectal coils were used in only 2 studies (25, 26), and all other studies used the phased-array coil (PAC). The majority of studies used mpMRI sequences of diffusion-weighted imaging (DWI), dynamic contrast-enhanced (DCE), and T2; however, 1 study used biparametric MRI (bpMRI) that only included DWI and T2 sequences (33).

**Table 1:** Demographic characteristics.

Study	Country	Year	Period	Patient Number	Lesion	Malignant	Age (year, mean±SD/median)	PSA (ng/ml, mean±SD/median)	Location
**Tamada et al.**	Japan	2019	2018.08–2019.03	58	58	26	69.7 (45-87)	8.07±4.98	TZ
**Wei et al.**	China	2020	2017.01–2020.03	355	355	93	69 (63-74)/ 73 (66-78)	9.55 (6.21-14.70)/ 13.65 (8.85-30.71)	TZ
**Kim et al.**	Korea	2020	2018.01–2018.12	317	317	103	64.0 (59.0–69.3)	4.9 (3.7–7.2)	PZ
**Yang et al.**	China	2020	2017.01–2017.12	159	159	30	70±8	NA	TZ
**Byun et al.**	Korea	2020	2018.01–2018.06	142	201	83	67 (46–81)	8.33+-7.81	TZ
**Lim et al.**	Canada	2020	2015.01–2018.07	109	109	35	64.8±8.4	10.6±7.2	TZ
**Urase et al.**	Japan	2021	2017.07–2019.12	77	616	228	68.3 ± 5.78	8.77 ± 5.27	TZ/PZ
**Brembilla et al.**	Italy	2020	2017.05–2017.09	200	200	61	65 (58–70)	6.0 (4.1–8.4)	Whole
**Bhayana et al.**	USA	2021	2015.11–2019.11	80	80	46	66/60-72	7.01/4.89-10.49	TZ/PZ
**Brancato et al.**	Italy	2020	2013.04–2018.09	111	117	78	69 (50–81)	0.26	Whole
**Hötker et al.**	Switzerland	2020	2015.01–2017.12	229	229	147	63.1 (46–79)	8.2/0.81–100	Whole
**Bao et al.**	China	2020	2018.01–2019.12	638	638	319	69 (53–95)	NA	Whole

NA, not available; PSA, prostate serum antigen; PZ, peripheral zone; TZ, transitional zone.

**Table 2:** Study and technical characteristics.

First Author	PI-RADS Version	No of Readers	Experience(Years)	Magnet Field Strength	*b* Values(mm^2^/sec)	Coil	Blinded	Sequence	Analysis	*κ* Value(95% CI)	Reference Standard
**Tamada**	2.1/2.0	2	7-12	3.0 T	0/1000/2000	PAC	Yes*	T2/DCE/DWI	Per Patient	0.65 (0.49-0.80)	TRUS+MRI-TRUS
**Wei**	2.1/2.0	5	1-8	3.0 T	0/100/1000/2000	PAC	Yes	T2/DWI	Per Patient	0.70 (0.65-0.75)	TRUS+MRI-TRUS
**Kim**	2.1/2.0	2	1-8	3.0 T	0/100/1000	PAC	Yes	T2/DCE/DWI	Per Patient	0.46 (0.36-0.56)	TRUS+MRI-TRUS
**Yang**	2.1/2.0	2	4-8	3.0 T	0/100/1000/2000	PAC	Yes	T2/DCE/DWI	Per Patient	0.81 (0.74-0.88)	TRUS
**Byun**	2.1/2.0	3	>3	3.0 T	0/500/1000/1500	PAC	Yes	T2/DCE/DWI	Per lesion	0.67 (0.60-0.74)	RP
**Lim**		3	2-7	3.0 T	0/500/1000	PAC	Yes	T2/DCE/DWI	Per Patient	0.55 (0.46-0.64)	MRI-TRUS
**Urase**	2.1/2.0	4	2-20	3.0 T	0/1000/2000	Cardiac	Yes	T2/DCE/DWI	Per lesion	0.64 (0.61-0.67)	RP
**Brembilla**	2.1	7	2-8	1.5 T	50/800/1600	ERC	Yes*	T2/DCE/DWI	Per Patient	0.59 (0.53-0.65)	TRUS
**Bhayana**	2.1	6	1-5	3.0 T	NA	PAC	Yes	T2/DCE/DWI	Per Patient	0.42 (0.32-0.52)	MRI-TRUS+RP
**Brancato**	2.1	3	7-10	1.5 T	50/400/1000/1400	ERC+PAC	Yes	T2/DCE/DWI	Per lesion	0.89 (0.84-0.94)	TRUS+MRI-TRUS
**Hötker**	2.1/2.0	2	2-6	3.0 T	100/600/1000	PAC	Yes	T2/DCE/DWI	Per Patient	0.52 (0.44–0.59)	TRUS
**Bao**	2.1	4	3-15	3.0 T	50/700/1500/2000	PAC	Yes	T2/DCE/DWI	Per Patient	0.82 (0.78-0.86)	TRUS+MRI-TRUS

ERC, endorectal coil; PAC, phased-array coil; DCE, dynamic contrast enhanced; DWI, diffusion weighted imaging; PI-RADS, Prostate Imaging Reporting and Data System; T2, T2 weighted imaging; TRUS, transrectal ultrasonography–guided biopsy; RP, radical prostatectomy; NA, not available.

*Aware of the patients’ age and PSA levels.

### Quality assessment

In general, the quality assessment for included studies was high. In 10 of 12 studies the readers were completely blinded to the final results, for the remaining 2 studies the radiologists were aware of part of the patient’s information (age and/or PSA level) that may influence the inter-reader agreement (26, 31). 5 studies did not explicitly report that the images were interpreted by readers independently, thus were categorized as high risk (24, 26, 32, 33, 35). Further details on the study quality are provided in **Supplementary Table 1**.

### Pooled inter-reader agreement of PI-RADS v2.1

**Figure 2:**
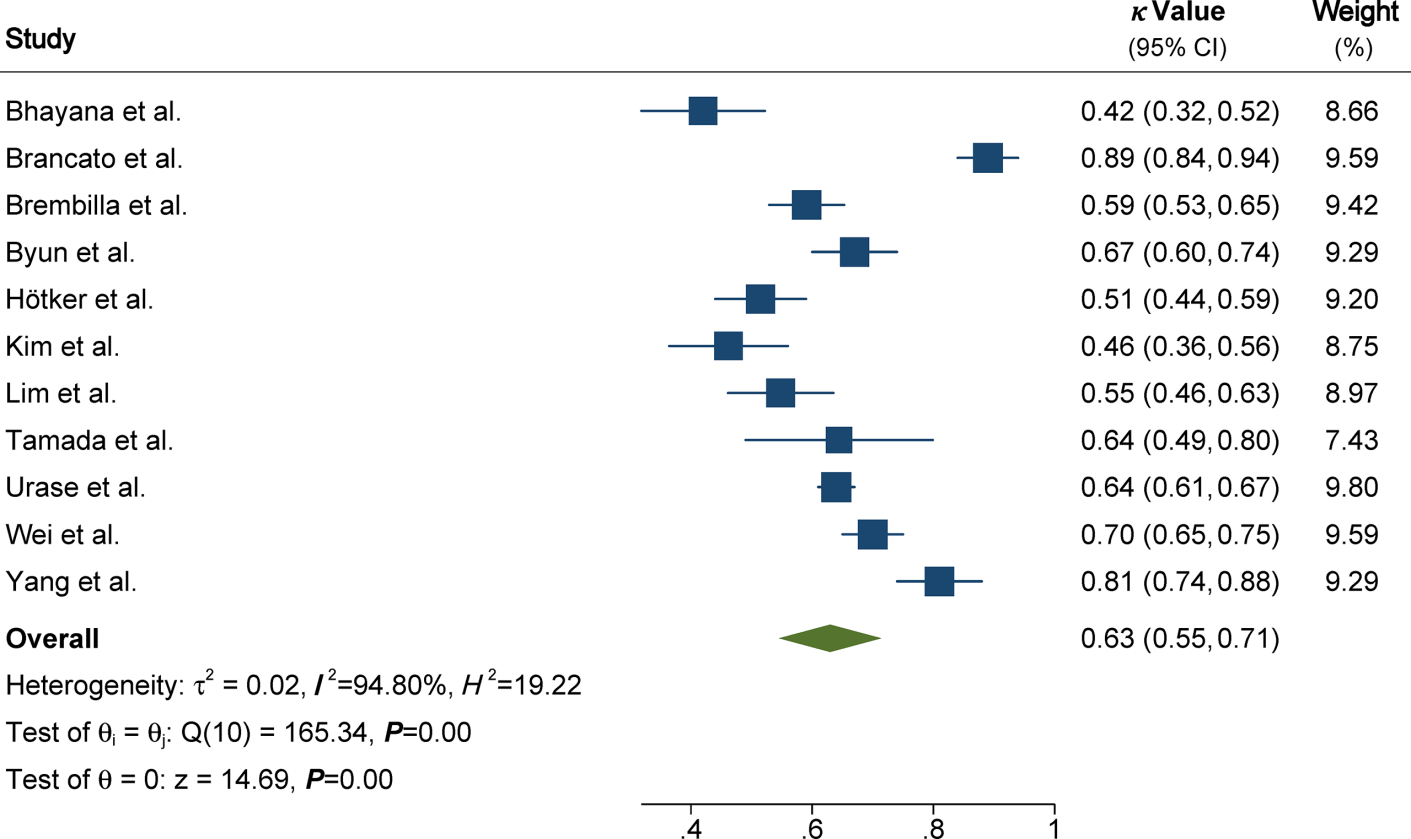
Coupled forest plot of pooled inter-reader agreement of all Prostate Imaging Reporting and Data System Version v2.1 lesions. CI, confidence interval.

**Figure 3:**
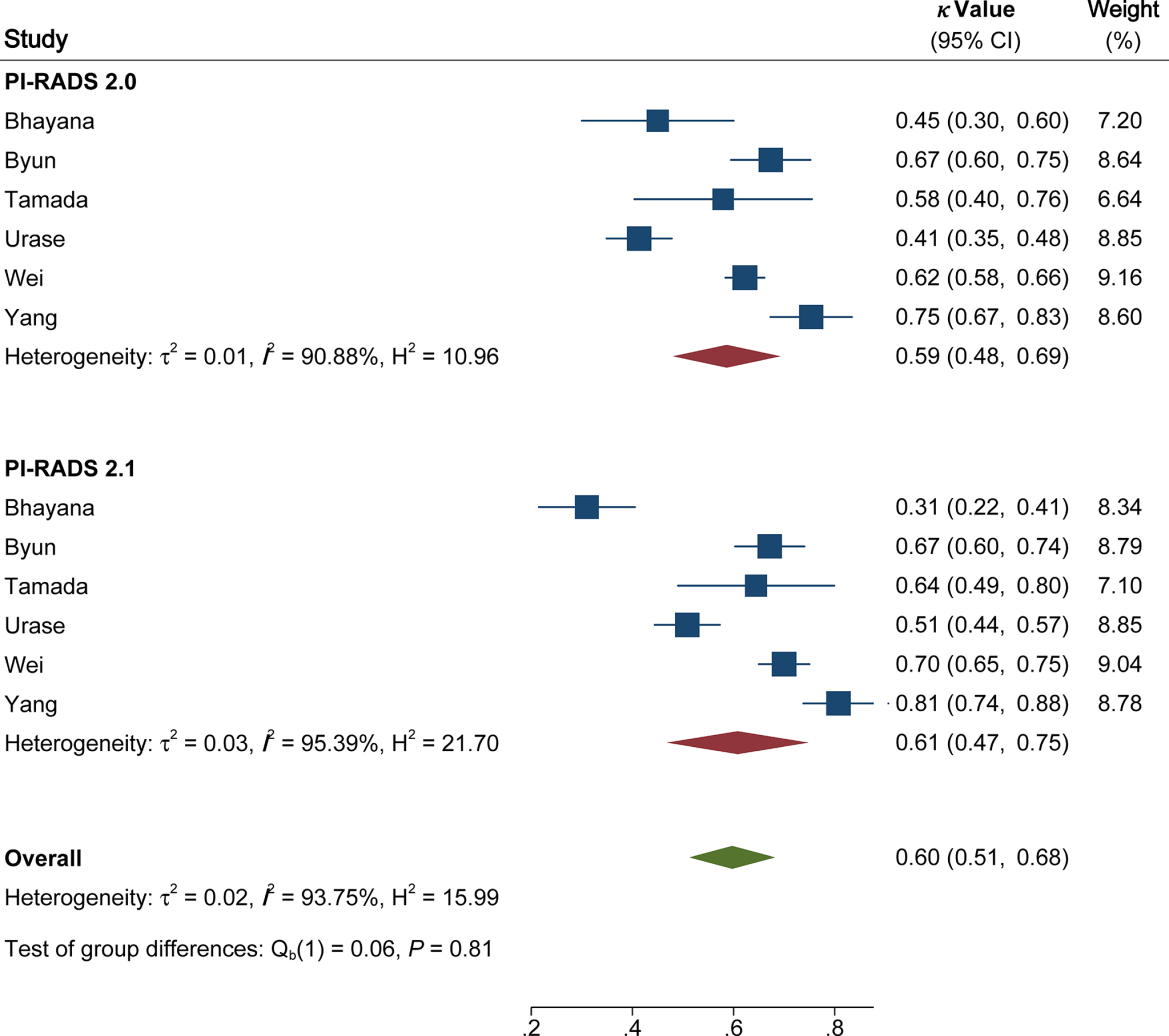
Coupled forest plot of pooled inter-reader agreement of Prostate Imaging Reporting and Data System Version v2.1 vs. 2.0 for transitional zone. CI, confidence interval.

**Figure 4:**
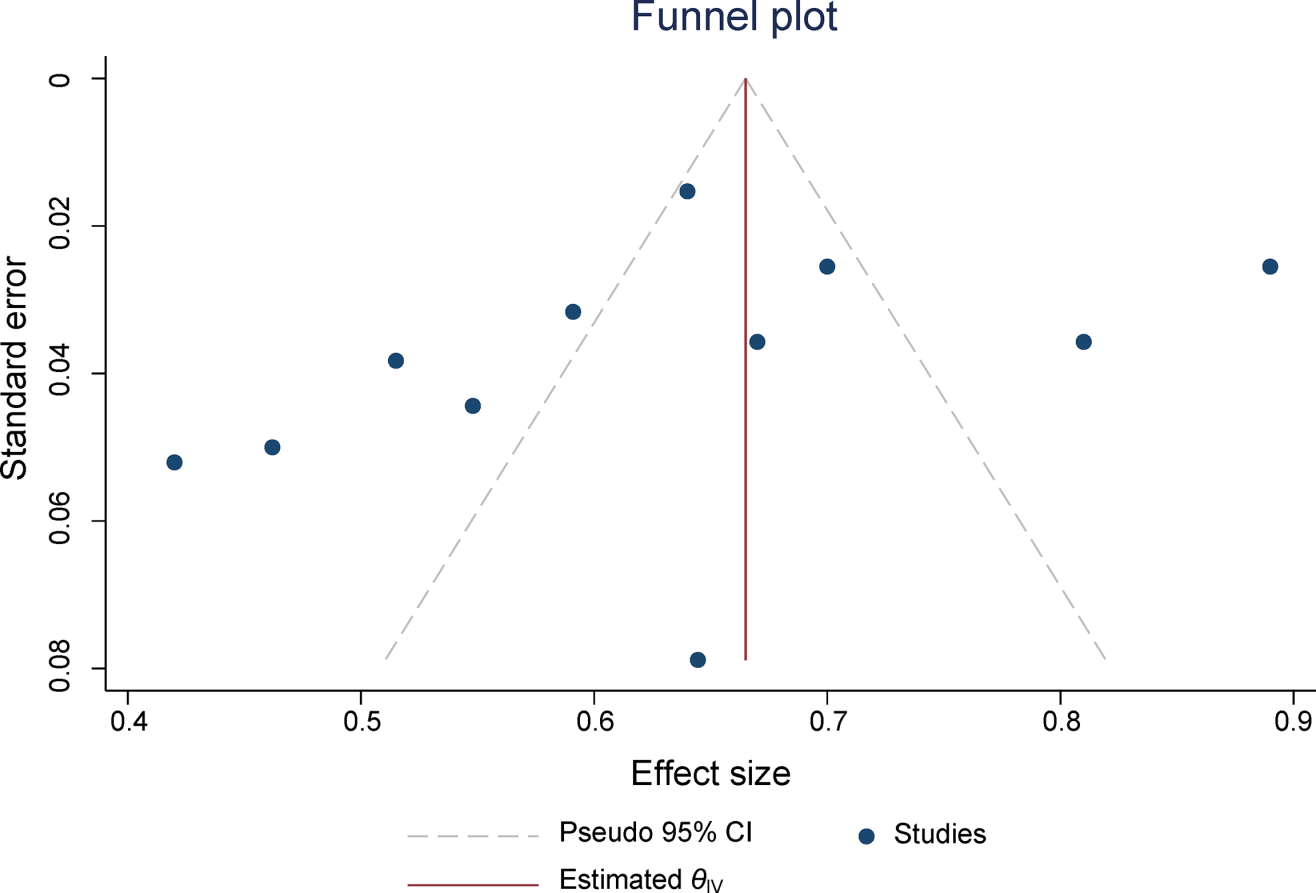
The funnel plot. A *P* value of 0.17 suggests that the likelihood of publication bias is low.

The pooled summary estimates of inter-reader agreement of the PI-RADS v2.1 are summarized in **Figure 2**. For individual studies, the *κ* values ranged from 0.42 to 0.89, and the pooled summary estimates of the *κ* value was 0.65 (95% CI 0.56-0.73) for all PI-RADS 1-5 lesions. We performed comparison between PI-RADS v2.0 and v2.1 using the available head-to-head comparison studies. For TZ lesions, the pooled *κ* value of 0.59 (95% CI 0.48-0.69) vs. 0.61 (95% CI 0.47-0.75) from 6 head-to-head comparison studies, suggesting the inter-reader agreement was comparable between the two PI-RADS versions (P=0.81, **Figure 3**) (24, 27, 31–34). Concerning the inter-reader agreement of all PI-RADS 1-5 lesions, the pooled *κ* values from 7 head-to-head comparison studies demonstrated that PI-RADS v2.1 had a non-significant difference compared with v2.0 with values of 0.64 (95% CI, 0.55-0.72) vs. 0.57 (95% CI, 0.45-0.69), respectively (p=0.37). We did not observe significant publication bias among included studies (**Figure 4**). Concerning the diagnostic accuracy for clinically significant PCa, the pooled sensitivity and specificity were 0.89 (95% CI 0.84-0.93) and 0.76 (95% CI 0.64, 0.85), with area under receiver operating characteristic (ROC) curve of 0.91 (95% CI 0.88-0.93).

### Multiple subgroup and meta-regression analysis

Concerning specific PI-RADS cutoff values, the pooled *κ* value was moderate (0.58, 95% CI 0.52-0.64) for PI-RADS score ≥3. Whereas for the threshold of PI-RADS score ≥4, the inter-reader agreement was substantial, with pooled *κ* value of 0.70 (95% CI 0.63-0.77). For the subgroup of any PCa, the pooled *κ* value was 0.67 (95% CI 0.55-0.79), which was comparable with the inter-reader agreement for clinically significant PCa (0.65, 95% CI 0.53-0.77). In terms of TZ lesions, the pooled inter-reader agreement from 8 studies was 0.62 (95% CI 0.51-0.72). By comparison, the inter-reader agreement for PZ lesions was slightly higher, with pooled *κ* value of 0.65 (95% CI 0.51-0.80). Regarding subgroup analysis according to experience, the pooled *κ* value for experienced readers (≥5 years) was not significantly different from inexperienced readers, with values of 0.72 (95%CI 0.66-0.78) and 0.64 (95% CI 0.60-0.68), respectively. For subgroup analysis of reference standard, the pooled *κ* value for TRUS was 0.62 (95% CI 0.49-0.75) for 4 studies, which was lower than 6 studies using MRI-TRUS as the reference standard (0.66, 95% CI 0.51-0.81).

As substantial heterogeneity was observed between included studies, we performed meta-regression analysis to investigate the sources. Among the various potential factors, we found that only the experience of radiologists (0.76 vs. 0.56, P<0.01) was significantly associated with heterogeneity. Other covariates such as type of analysis (per patient vs. per lesion), PI-RADS score (lesions with score ≥3 vs. all lesions), and zonal anatomy (whole gland vs. TZ) were not substantial factors, with P values ranging from 0.14 to 0.43 (**Supplementary Table 2**).

## Discussion

The inter-reader agreement is critical for the standardized scoring system, as it relates to reducing the variability of interpretation between radiologists and improving the categorization of the level of suspicion for PCa. In this study, we systematically assessed the inter-reader agreement of PI-RADS v2.1 for the detection of PCa based on 12 studies. The pooled *κ* value of 0.65 (95% CI 0.56-0.73) suggested that the PI-RADS v2.1 exhibited substantial reproducibility among radiologists. Such reliability is critical for a standardized scoring system, as it reduces variable interpretation among radiologists and improves the association of categorization with likelihood of clinically significant PCa. As the revision of PI-RADS v2.1 served a main purpose of improving the inter-reader agreement for TZ lesions, the majority of included studies assessed the reproducibility for lesions located in TZ. Nevertheless, the pooled *κ* values suggested that there was no significant improvement as compared to PI-RADS v2.0 in several head-to-head comparison studies. Furthermore, half of the included studies reported no improvement of inter-reader agreement or even a decrease, though findings may have been at least partially affected by experience of readers (24, 25, 27).

In this meta-analysis substantial heterogeneity was observed between included studies, hence meta-regression analysis was performed to investigate the sources. Among the various potential factors, we found that only the level of readers’ experience was significantly associated with the degree of heterogeneity (P<0.01). In some studies, the *κ* value was generated from readers of widely varied experience, which is usually lower than those from experienced readers (24, 26, 28–30). Another possible but less studied influence on reader agreement is that of image quality (e.g. high ***b*** value images of DWI), as clear, quantifiable quality standards are not established (15). The meta-regression is considered exploratory and there may have been too few head-to-head studies to detect a difference in heterogeneity according to PI-RADS version. Our analysis revealed that the inter-reader agreement was lower in PI-RADS score ≥3 lesions relative to all lesions (0.58 vs. 0.65), one possible explanation is that more detailed diagnostic criteria of PI-RADS 1-2 score introduced in v2.1. On the other side, the inter-reader agreement for PI-RADS ≥4 was substantial, with a pooled *κ* value of 0.7. Therefore, the classification of score 3 lesions still a challenge in the new PI-RADS version, which may more depend on readers’ experience and personal patterns, and future studies may focus on more quantitative means of scoring. Although studies demonstrated that bpMRI has comparable diagnostic accuracy as multiparametric MRI in the detection of PCa (36), the majority of studies included the DCE sequence. In 3 studies that provided the inter-reader agreement from bpMRI, the reported pooled *κ* value of 0.7-0.86 suggested substantial to good agreement among readers. Because of too small sample, the *κ* values were unfeasible to pool and need validation in large prospective multi-center studies in the future.

In an earlier meta-analysis conducted by Park et al., the pooled *κ* value for PI-RADS v2 was 0.61 (95% CI 0.55-0.67) based on 30 studies, which is slightly lower than our results (15). In a more recent study, the reported *κ* values for PI-RADS v2.0 and v2.1 were 0.42-0.70 and 0.48-0.69, respectively, which also demonstrated comparable inter-reader agreement (37). Although many modifications in PI-RADS v2.1 were related to TZ lesions, some important revisions were also introduced to the interpretation of PZ lesions. On the basis of 5 studies reporting the inter-reader agreement on PZ lesions (24–26, 29, 32), the pooled *κ* value was comparable to a previous study (0.65 vs. 0.64) (15). In general, our study indicated that there was no substantial improvement in reproducibility between readers based on current evidence. Recently, radiomics has been widely studied in PCa, with investigations between quantitative image features and single gene expression, which demonstrated promising diagnostic results. In addition, artificial intelligence and machine learning also have been thoroughly studies in management and treatment of PCa such as biopsy, surgery, histopathology, and active surveillance (38, 39).

There are some limitations to our study. First, all studies included were retrospective in the study design, leading to a high risk of bias for the patient selection domain. Nevertheless, because of insufficient data it is unfeasible to pool the inter-reader agreement from prospective studies. Second, substantial heterogeneity was presented among included studies, which affected the general applicability of this systematic review. We performed multiple subgroup analyses and meta-regression to explore the sources, and the results indicated that level of experience was the significant factor that contributed to the heterogeneity. However, these analyses only explained part of the heterogeneity, and these analyses were based on only a few studies, thus the results should be interpreted cautiously. Lastly, most included studies used MRI-TRUS fusion targeted biopsy as the reference standard, while compared to RP which may miss potential lesions with a negative MRI but positive pathology.

## Conclusion

PI-RADS v2.1 demonstrated substantial inter-reader agreement between radiologists for whole gland and TZ lesions. Nevertheless, no statistically significant improvement in reproducibility was observed in PI-RADS v2.1 of as compared to v2.0.

## Data availability statement

The original contributions presented in the study are included in the article/**Supplementary Material**. Further inquiries can be directed to the corresponding author.

## Author contributions

All authors listed have made a substantial, direct, and intellectual contribution to the work, and approved it for publication.

## Conflict of interest

The authors declare that the research was conducted in the absence of any commercial or financial relationships that could be construed as a potential conflict of interest.

## Publisher’s note

All claims expressed in this article are solely those of the authors and do not necessarily represent those of their affiliated organizations, or those of the publisher, the editors and the reviewers. Any product that may be evaluated in this article, or claim that may be made by its manufacturer, is not guaranteed or endorsed by the publisher.
