# Supplementary material for: Inter-reader agreement of the prostate imaging reporting and data system version v2.1 for detection of prostate cancer: A systematic review and meta-analysis
Source: Front Oncol. 2022 Sep 29;12:1013941. doi: 10.3389/fonc.2022.1013941 (PMC9554626; doi:10.3389/fonc.2022.1013941)
Supplement: Supplementary file 1 [file Table_1.docx]

**Supplementary Table 1.** Risk of bias assessment according to Quality Appraisal of Diagnostic Reliability (QAREL) Checklist.

| **Study** | **Year** | **Item 1** | **Item 2** | **Item 3** | **Item 4** | **Item 5** | **Item 6** | **Item 7** | **Item 8** | **Item 9** | **Item 10** | **Item 11** |
| --- | --- | --- | --- | --- | --- | --- | --- | --- | --- | --- | --- | --- |
| Tamada et al. | 2019 | + | + | high | ? | + | + | - | NA | NA | + | + |
| Wei et al. | 2020 | + | + | ? | high | + | + | ? | NA | NA | + | + |
| Kim et al. | 2020 | + | + | high | high | + | + | + | NA | NA | + | + |
| Yang et al. | 2020 | + | + | high | high | + | + | + | NA | NA | + | + |
| Byun et al. | 2020 | + | + | high | high | + | + | + | NA | NA | + | + |
| Lim et al. | 2020 | + | ? | high | high | + | + | + | NA | NA | + | + |
| Urase et al. | 2021 | + | + | ? | high | + | + | ? | NA | NA | + | + |
| Brembilla et al. | 2020 | + | + | ? | ? | + | + | - | NA | NA | + | + |
| Bhayana et al. | 2021 | + | + | ? | high | + | + | - | NA | NA | + | + |
| Brancato et al. | 2020 | + | + | high | high | + | + | + | NA | NA | + | + |
| Hötker et al. | 2020 | + | + | high | high | + | + | + | NA | NA | + | + |
| Bao et al. | 2020 | + | + | ? | high | + | + | ? | NA | NA | + | + |

1. Was the test evaluated in a sample of subjects who were representative of those to whom the authors intended the results to be applied?

2. Was the test performed by raters who were representative of those to whom the authors intended the results to be applied?

3. Were raters blinded to the findings of other raters during the study?

4. Were raters blinded to their own prior findings of the test under evaluation?

5. Were raters blinded to the results of the reference standard for the target disorder (or variable) being evaluated?

6. Were raters blinded to clinical information that was not intended to be provided as part of the testing procedure or study design?

7. Were raters blinded to additional cues that were not part of the test?

8. Was the order of examination varied?

9. Was the time interval between repeated measurements compatible with the stability (or theoretical stability) of the variable being measured?

10. Was the test applied correctly and interpreted appropriately?

11. Were appropriate statistical measures of agreement used?

NA: not applicable.
