# Supplementary material for: Inter-reader agreement of the prostate imaging reporting and data system version v2.1 for detection of prostate cancer: A systematic review and meta-analysis
Source: Front Oncol. 2022 Sep 29;12:1013941. doi: 10.3389/fonc.2022.1013941 (PMC9554626; doi:10.3389/fonc.2022.1013941)
Supplement: Supplementary file 2 [file Table_2.docx]

**Supplementary Table 2. Meta-Regression**

| Covariate | | Kappa | Recalculated ***I***^2^ | *P* Value |
| --- | --- | --- | --- | --- |
| Zonal anatomy | Whole | 0.62/0.48-0.75 | 97.84% | 0.43 |
|  | TZ | 0.68/0.59-0.76 | 83.34% |  |
| Analysis | Per patient | 0.62/0.52-0.71 | 93.75% | 0.2 |
|  | Per lesion | 0.73/0.58-0.89 | 96.77% |  |
| Reader Experience | Varied experience | 0.56/0.49-0.63 | 89.43% | <0.01 |
|  | Experienced | 0.78/0.69-0.87 | 89.53% |  |
| PI-RADS Score | All | 0.68/0.57-0.79 | 96.35% | 0.14 |
|  | ≥3 | 0.57/0.49-0.66 | 76.29% |  |

**Subgroup Analysis**

| **Item** | **Kappa (95% CI)** |
| --- | --- |
| PI-RADS ≥3 | 0.58/0.52-0.64 |
| PI-RADS ≥4 | 0.70/0.63-0.77 |
| Whole Gland | 0.66/0.50-0.81 |
| TZ | 0.60/0.50-0.71 |
| PZ | 0.65/0.51-0.80 |
| Experienced | 0.72/0.66-0.78 |
| Inexperienced | 0.64/0.60-0.68 |
| CS PCa | 0.65/0.53-0.77 |
| Any PCa | 0.67/0.55-0.79 |
